# Genomic surveillance of *Neisseria meningitidis* serogroup B invasive strains: Diversity of vaccine antigen types, Brazil, 2016-2018

**DOI:** 10.1371/journal.pone.0243375

**Published:** 2020-12-21

**Authors:** Ana Paula Silva de Lemos, Claudio Tavares Sacchi, Claudia Regina Gonçalves, Carlos Henrique Camargo, Ana Lúcia Andrade

**Affiliations:** 1 Centre of Bacteriology, Institute Adolfo Lutz (IAL), São Paulo, Brazil; 2 Strategic Laboratory, Institute Adolfo Lutz (IAL), São Paulo, Brazil; 3 Institute of Tropical Pathology and Public Health, Federal University of Goiás, Goiânia, Brazil; Cornell University, UNITED STATES

## Abstract

**Background:**

*Neisseria meningitidis* serogroup B remains a prominent cause of invasive meningococcal disease (IMD) in Brazil. Because two novel protein-based vaccines against serogroup B are available, the main purpose of this study was to provide data on the diversity and distribution of meningococcal vaccine antigen types circulating in Brazil.

**Methodology:**

Genetic lineages, vaccine antigen types, and allele types of antimicrobial-associated resistance genes based on whole-genome sequencing of a collection of 145 *Neisseria meningitidis* serogroup B invasive strains recovered in Brazil from 2016 to 2018 were collected.

**Results:**

A total of 11 clonal complexes (ccs) were identified among the 145 isolates, four of which were predominant, namely, cc461, cc35, cc32, and cc213, accounting for 72.0% of isolates. The most prevalent fHbp peptides were 24 (subfamily A/variant 2), 47 (subfamily A/variant 3), 1 (subfamily B/variant 1) and 45 (subfamily A/variant 3), which were predominantly associated with cc35, cc461, cc32, and cc213, respectively. The NadA peptide was detected in only 26.2% of the isolates. The most frequent NadA peptide 1 was found almost exclusively in cc32. We found seven NHBA peptides that accounted for 74.5% of isolates, and the newly described peptide 1390 was the most prevalent peptide exclusively associated with cc461. Mutated *penA* alleles were detected in 56.5% of the isolates, whereas no *rpoB* and *gyrA* mutant alleles were found.

**Conclusion:**

During the study period, changes in the clonal structure of circulating strains were observed, without a predominance of a single hyperinvasive lineage, indicating that an epidemiologic shift has occurred that led to a diversity of vaccine antigen types in recent years in Brazil.

## Introduction

Invasive meningococcal disease (IMD) remains a major health problem and is a major cause of morbidity and mortality worldwide. Globally, most IMD cases are caused by serogroups A, B, C, W, Y, and X [1].

In the past three decades, IMD in Brazil has been primarily caused by meningococcal strains of serogroups B and C. During the 2000s, the proportion of IMD cases caused by serogroup B meningococcal strains declined from 74.5% in 2000 to 25.7% in 2008, corresponding to a decline from 0.54 to 0.14 per 100,000 inhabitants in the average annual incidence of disease due to this serogroup (S1 Fig). At the same time, serogroup C IMD cases increased from 22.4% to 67.7% at the national level, except for in the South Region, where serogroup C IMD surpassed serogroup B only in 2014 [2]. Indeed, the annual incidence of serogroup C IMD increased from 0.2 cases in 2000 to 0.46 cases per 100,000 habitants in 2008, making C the prevalent serogroup circulating in Brazil from 2005 onward (S1 Fig). In late 2010, the routine meningococcal C conjugate (MCC) vaccination was introduced in the National Immunization Program (NIP), which targeted only infants, and MCC has been included in the routine immunization calendar for adolescents since 2017. After its introduction by the NIP, MCC vaccination was considered to be highly effective in Brazil based on the reduction of IMD caused by serogroup C in infants [3]. Although we observed a downward trend of IMD following the introduction of the MCC vaccine, from 1.54 per 100,000 in 2010 to 0.54 per 100,000 habitants in 2018, and observed a significant declining trend in incidence rates of serogroup B IMD cases [4] (S1 Fig), meningococcal serogroup B (MenB) remains the prominent cause of IMD in Brazil, particularly in infants, with 1.5 cases per 100,000 habitants in 2018 (S2 Fig).

The structural similarity between the serogroup B polysaccharide and fetal brain-cell adhesion molecules makes the B polysaccharide a poor immunogen [5]. This similarity reduces the ability to elicit bactericidal antibodies and raises concerns about possible autoimmunity. Therefore, several noncapsular outer membrane vesicle vaccines were used against *Neisseria meningitidis* serogroup B, and vaccines based on recombinant proteins identified by proteomic and reverse vaccinology approaches are currently available [6, 7].

Since late 2013, two novel protein-based vaccines against serogroup B have been licensed in Europe and the Americas (MenB-FHbp, Trumenba, Pfizer, and 4CMenB, Bexsero, GlaxoSmithKline). The MenB-FHbp vaccine is composed of two recombinant lipidated factor H-binding proteins (fHbp) from subfamilies A (A05) and B (B01). The 4CMenB multicomponent vaccine is composed of multiple recombinant antigens: a nonlipidated fHbp peptide 1 from subfamily B/variant 1, Neisserial adhesin A (NadA) peptide 8 variant NadA-2/3, Neisserial heparin-binding antigen (NHBA) peptide 2 and PorA P1.7–2,4 from the New Zealand epidemic strain [8, 9]. Studies in different countries have detailed the distribution and diversity of these vaccine proteins [10–16]. Since 2015, the Men-FHbp and 4CMenB vaccines have been implemented in 12 countries in children and adolescents in NIPs [17–19], for the control of outbreaks in the United States and Canada [20, 21] or individual clinical decisions in the United States [20].

Nonetheless, data on the distribution and diversity of these vaccine antigens in Latin America are scarce [13, 22, 23]. Therefore, we aimed to evaluate the genetic lineages, the diversity of vaccine antigens, and the alleles types of antimicrobial resistance-associated genes in serogroup B IMD strains isolated in Brazil from 2016 to 2018 using whole-genome sequencing. The ultimate goal was to support the NIP in the future decision to implement a MenB vaccination.

## Material and methods

### Collection of strains

#### Epidemiological data and bacterial isolates

During the period from 2016 to 2018, 3,317 IMD cases were reported to the Ministry of Health in Brazil from all geographic regions of the country [2]. IMD isolates collected by the Brazilian meningococcal surveillance network are routinely sent to Adolfo Lutz Institute (IAL)–the National Reference Laboratory for serogrouping and other tests. A total of 2,518 (76.0%) of 3,317 cases reported between 2016 and 2018 were confirmed in the IAL by either (i) culture (882 cases; 35.0%), (ii) detection of meningococcal DNA in cerebrospinal fluid, serum, or tissue by quantitative polymerase chain reaction (qPCR) (935 cases; 37.1%), (iii) detection of meningococcal antigens in cerebrospinal fluid or serum by counterimmunoelectrophoresis and/or latex agglutination (401 cases; 16.0%), or (iv) identification of the presence of gram-negative diplococci by Gram staining of cerebrospinal fluid (300 cases; 11.9%). Of the 2,218 cases confirmed in the IAL by culture and antigen or DNA detection, the serogroup was determined for 1,536 cases (69.2%), with 403 cases (26.2%) caused by serogroup B. In this study, we analyzed the genetic profile of 145 serogroup B IMD isolates received at the IAL from 2016 to 2018 (S1 Table).

### Laboratory procedures

#### Extraction of DNA

Genomic DNA extraction and purification were performed using the QIAamp DNA Mini Kit (Qiagen, Hilden, Germany) according to the manufacturer’s instructions.

#### Whole-genome sequencing

Genome library preparation was performed using the Ion Xpress Plus Fragment Kit (Thermo Fisher Scientific, Waltham, MA, USA), and genome libraries corresponding to 400 base pairs were prepared using E-Gel SizeSelect Agarose Gel, 2% (Thermo Fisher Scientific, Waltham, MA, USA). Genome sequencing was performed using the Ion Torrent S5 sequencer (Thermo Fisher Scientific, Waltham, MA, USA), and the reads were assembled *de novo* using SPAdes v.5.6.0.1.

#### Genome analysis

The assembled contigs were functionally annotated by the NCBI Prokaryotic Genome Annotation Pipeline. Sequence assignment was performed using the gene-by-gene approach with the PubMLST *Neisseria* database (https://pubmlst.org/neisseria/).

Nucleotide sequences of *fHbp*, *nadA*, and *nhba* were extracted from fasta files, converted to deduced protein sequences with BioNumerics v.7.6.2, and then aligned by using ClustalW (CLC Genomics Workbench 7). SplitsTree v.5.0 was employed to create phylogenetic networks. Genome reference sequence(s) were included for each antigen as follows: peptide 1 (B24/1.1; accession number NZ_AEQZ00000000.1), peptide 45 (A05/3.45; accession number AY330361.1), and peptide 55 (B01/1.55; accession number AY330406.01) for fHbp; peptide 8 (NadA-2/3; accession number GQ302859.1) for NadA; and peptide 2 for NHBA; accession number AF226445.1).

High-quality single nucleotide polymorphisms (SNPs) were called using the mpileup part of SAMTools v. 0.1.18 [24] from assembled genomes, and a phylogenetic tree based on the concatenated alignments was built in CSI Phylogeny 1.4 (https://cge.cbs.dtu.dk/services/CSIPhylogeny/) [25]. The genome of *Neisseria meningitidis* H44/76 (GenBank accession number CP002420.1) was included as a reference. The Newick generated file was uploaded to the Microreact platform [26] along with metadata for tree visualization.

#### Meningococcal characterization by multilocus sequence typing (MLST)

Multilocus sequence typing (MLST) enables the identification of meningococcal hyperinvasive lineages that are particularly associated with disease [27]. Arbitrary integer values are assigned to unique alleles at each of the defined seven housekeeping gene loci. Sequence types (STs) are unique combinations of these seven alleles from the defined locus. Groups of related STs were grouped into clonal complexes (ccs) identified by MLST [27].

#### Meningococcal scheme nomenclature for antigenic variants

Different classification schemes have been proposed to describe allelic variation in the *fhbp* gene and the corresponding peptides: the Novartis/GlaxoSmithKline nomenclature classifies the protein variants of fHbp into three variant families, variants 1, 2, and 3 [28]; the Pfizer nomenclature classifies it into subfamilies A and B [29]; and the fHbp database at PubMLST proposes a unified nomenclature in which unique fHbp peptide and nucleotide sequences are arbitrarily assigned sequential numbers in order of discovery independent of subfamily/variant [30]. For this study, a combination of these systems was used: subB/var1 correlates to subfamily B or variant 1, and subA/var2-3 correlates to subfamily or variants 2 and 3. The NadA classification is based on peptide sequence homology in one of the four variants described: NadA-1, NadA-2/3, NadA-4/5, and NadA-6 [31]. The NHBA peptide is assigned arbitrarily integer values to unique peptide sequences. PorA classification is based on nucleotide and peptide sequence homology and recognized the previous serologic classification: the prefix "P1." followed by the VR1 family name, followed by a dash and then the variant number, and the VR2 variant name in the same format [32]. All nomenclature is available on the PubMLST website (https://pubmlst.org/).

#### Statistical analysis

The association of fHbp subfamily/variant with patient age was evaluated using the chi-square test, and a *p*-value lower than 0.05 was used to denote statistical significance. Information on patient age is provided in S1 Table.

#### Data availability statement

Nucleotide sequences have been deposited in GenBank under the accession numbers listed in S2 Table.

This study was approved by the scientific (CTC 39-K/2018) and ethical committees (n. 3.448.268) of the Institute Adolfo Lutz (IAL), São Paulo, Brazil, and Institute of Tropical Pathology and Public Health, Federal University of Goiás, Goiânia, Brazil.

## Results

### Clonal complex distribution

**Fig 1:**
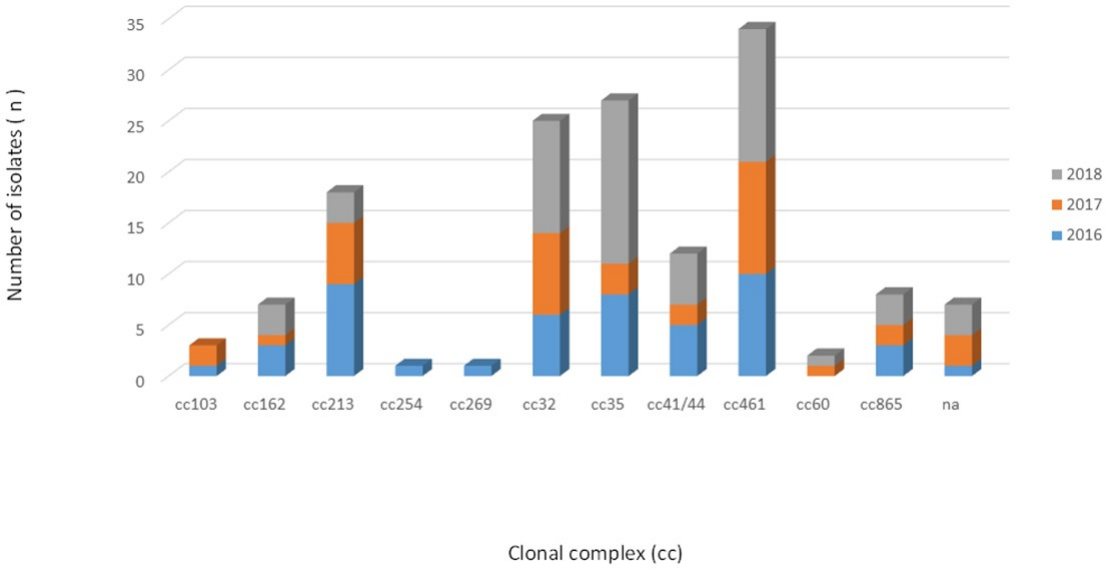
Distribution of clonal complexes among 145 serogroup B invasive meningococcal disease isolates, Brazil, 2016–2018.

By MLST, a total of 41 different STs were identified among the 145 isolates, of which 138 isolates could be assigned to 11 ccs: cc461 (n = 34/145; 23.4%), cc35 (n = 27/145; 18.6%), cc32 (n = 25/145; 17.2%), cc213 (n = 18/145; 12.4%), cc41/44 (n = 12/145; 8.3%), cc865 (n = 8/145; 5.5%), cc162 (n = 7/145; 4.8%), cc103 (n = 3/145; 2.1%), cc60 (n = 2/145; 1.4%), cc269 (n = 1/145; 0.7%), and cc254 (n = 1/145; 0.7%). Overall, 104 isolates (72.0%) were found to belong to cc213, cc32, cc35 and cc461 (Fig 1). The remaining 7 isolates, STs 1768, 4221, 14576, 14577, 14578, 14630 and 15345, were not assigned to ccs (S2 Table).

### Genetic variability and distribution of PorA, PorB, and FetA

Among 142 isolates, we found 17 alleles of PorA VR1 and 28 alleles of PorA VR2. A total of 31 different combinations of VR1/VR2 PorA were identified. For the remaining three isolates, no nucleotide sequence allele was assigned because of the presence of a premature stop codon. Five combinations, P1.19,15 (n = 25/145; 17.3%), P1.19,15–1 (n = 23/145; 15.9%), P1.22,14 (n = 17/145; 11.7%), P1.7–2,4 (n = 15/145; 10.3%) and P1.19–2,13–1 (n = 14/145; 9.6%), accounted for 64.8% (n = 94) of the isolates. The remaining combinations represented 5% or less of the isolates. In addition, 17 combinations were represented by only a single isolate. The above five combinations were strongly associated with the cc detected. P1.19 and 15 belong to cc32 (n = 21/25; 84.0%), P1.19 and 15–1 to cc35 (n = 20/23; 87.0%), P1.22 and 14 to cc213 (n = 16/18; 94.0%), P1.19–2 and 13–1 to cc461 (n = 14/34; 41.2%), and P1.7–2 and 4 to cc41/44 (n = 8/15; 53.0%) and cc162 (n = 7/15; 47.0%) (S3 Fig). PorA VR2 variant 4, included as an antigen in the 4CMenB vaccine, was found in 15 (10.3%) isolates and was associated exclusively with PorA VR1 variant 7–2 belonging to different ccs (cc41/44, n = 8/15; 53.0% and cc162, n = 7/15; 47.0%) (S2 Table).

Thirty-five alleles of PorB2 (n = 2) and PorB3 (n = 33) were found among the variants. The most prevalent alleles of PorB3 were 3–14 (30/145; 20.7%), 3–1 (29/145; 20.0%), and 3–45 (24/145; 16.6%), which were distributed in the five predominant ccs (32, 35, 41/44, 213 and 461) (S3 Fig). Some PorB3 alleles (985, 986, 987, 1010, 1011, 1012, 1013, 1014, 1015, 1016, 1190) had not been previously assigned (S2 Table).

Among 145 isolates, 17 alleles for FetA variants were grouped into four families of FetA. The most prevalent variants were F5-5 (n = 46/145, 31.7%), F1-7 (n = 31/145, 21.4%), F5-1 (n = 19/145, 13.1%), F1-5 (n = 12/145, 8.3%), and F5-9 (n = 11/145, 7.6%), which were distributed among the five predominant cc (32, 35, 41/44, 213 and 461) (S2 Table, S3 Fig). The remaining combinations represented 4% or less of the isolates. FetA variants F4-78 and F5-194 have not been previously assigned (S2 Table).

### Genetic variability and distribution of NadA

**Fig 2:**
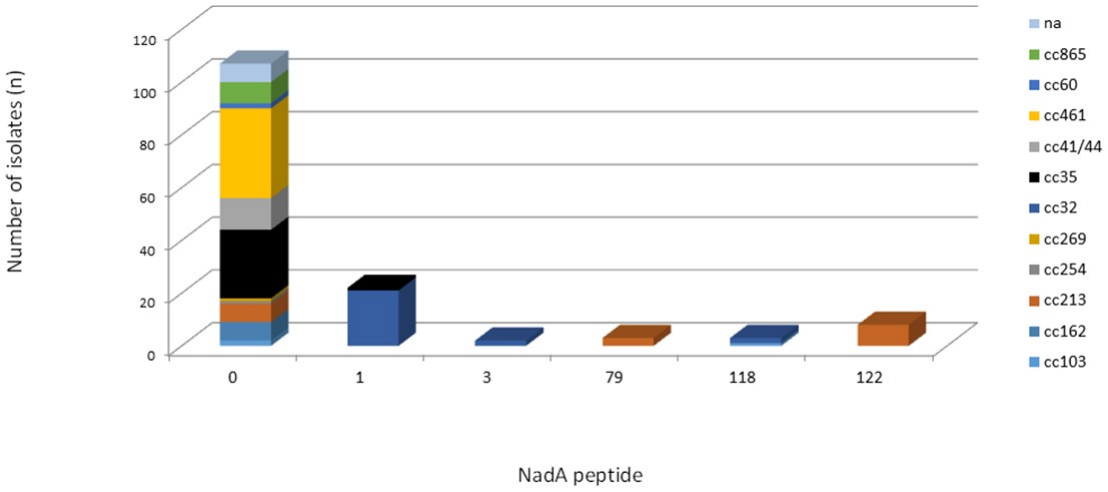
Frequency distribution of NadA peptides by clonal complex among 145 serogroup B invasive meningococcal disease isolates, Brazil, 2016–2018.

**Fig 3:**
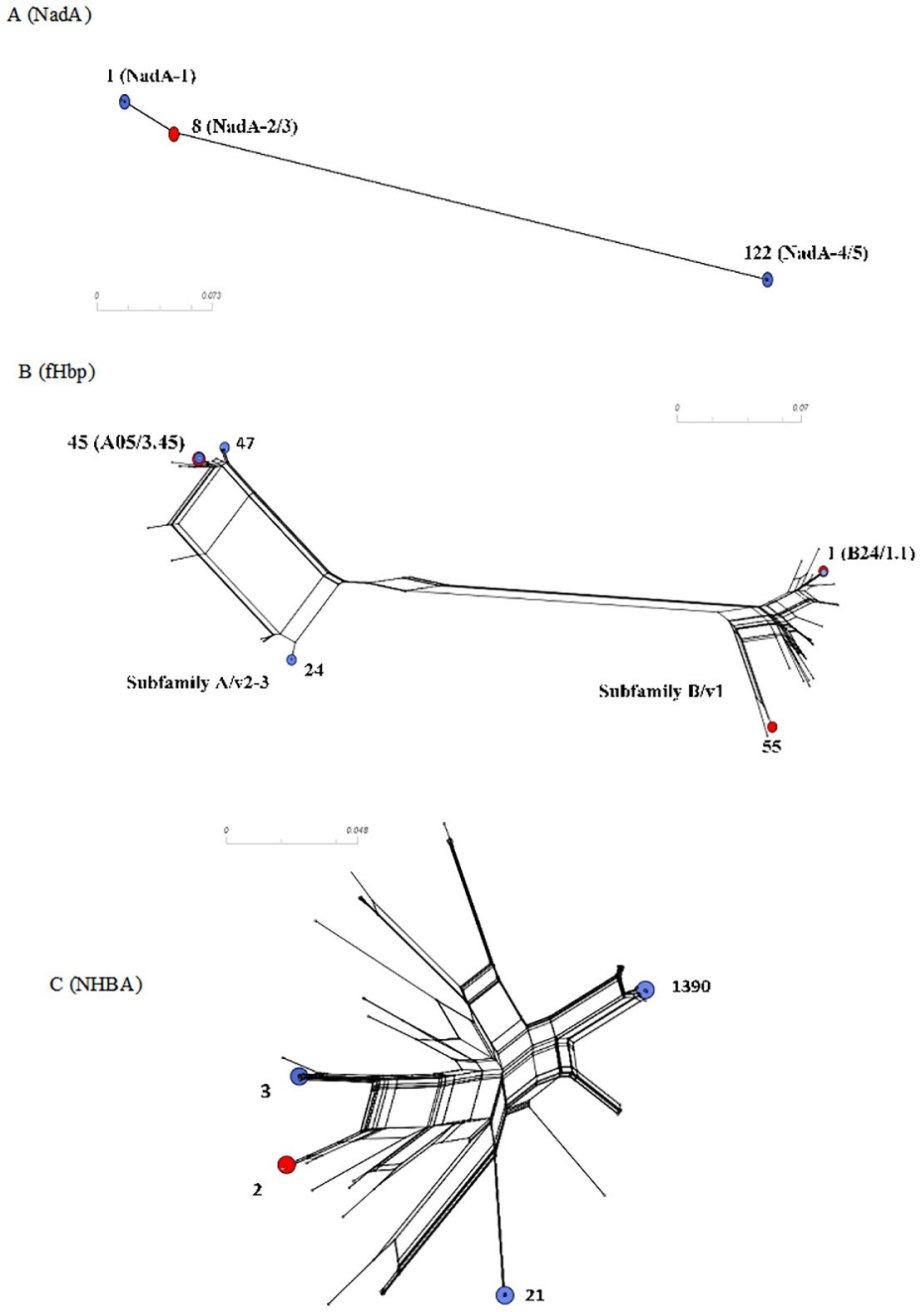
Phylogenetic analysis of (A) fHbp, (B) NadA, and (C) NHBA peptides. Reference peptide sequences fHbp 45 and 55 correspond to antigens in MenB-FHbp, and fHbp 1, NadA3-8, NHBA 2 correspond to antigens in 4CMenB vaccines. The reference peptides are indicated by red circles in the figures, and the prevalent ones are indicated by blue circles.

Most isolates (n = 107/145, 73.8%) were unable to present the potential for expression of the NadA peptide due to the absence of the *nadA* gene, the presence of frameshift mutations, or the presence of insertion elements. The NadA peptide was identified in only 38 (38/145, 26.2%) isolates. Among the 38 isolates, NadA peptides 1 and 118 were grouped as variant 1 (n = 25/38, 65.8%), peptide 3 as variant 2/3 (n = 2/38, 5.3%) and peptides 79 and 122 as variant 4/5 (n = 11/38, 28.9%). Four isolates were assigned to variant 4/5, but the nadA nucleotide allele (allele 34) did not predict the expression of any peptide due to the shifted reading frame. The NadA variants were mainly observed in cc213 (n = 15) and cc32 (n = 25). cc32 was the only lineage in which the isolates presented the potential to express NadA peptide. The NadA peptide was absent from all isolates belonging to cc162, cc254, cc269, cc41/44, cc461, cc60, and cc865 (Fig 2). The maximum pairwise amino acid identity among peptides between NadA-1 and NadA2-3 was 97.0%; between NadA-1 and NadA-4/5, it was 70.0%, and between NadA-2/3 and NadA-4/5, it was 73.0%. Additionally, the pairwise amino acid identity within NadA-1, NadA2-3, and NadA4-5 was at least 99.0%, 100.0% and 95.0%, respectively (Fig 3).

### Genetic variability and distribution of NHBA

**Fig 4:**
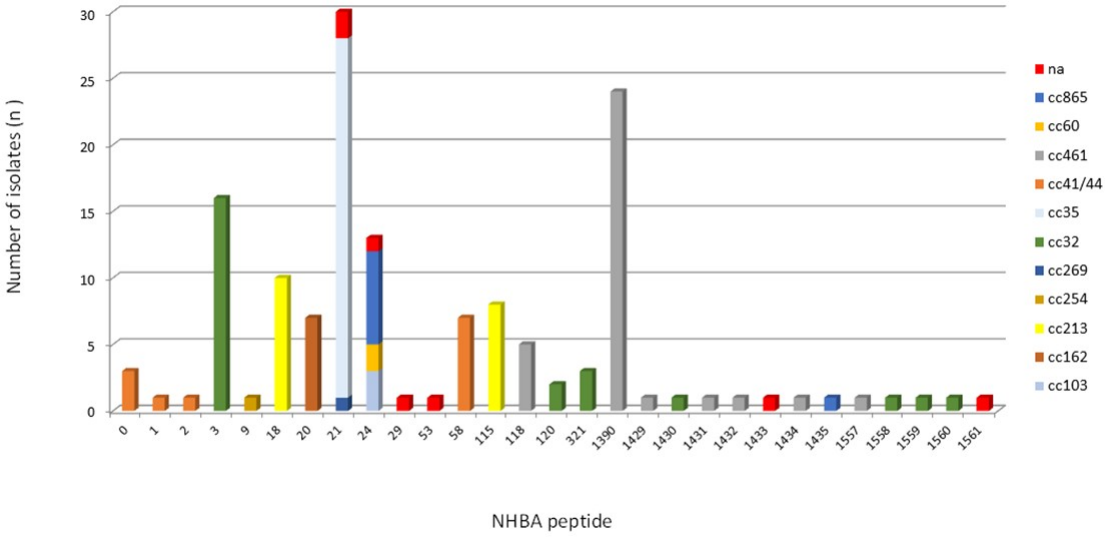
Frequency distribution of NHBA peptides by clonal complex among 145 serogroup B invasive meningococcal disease isolates, Brazil, 2016–2018.

Most of the isolates (n = 142/145, 98.0%) contained the intact *nhbA* gene, and three isolates were unable to present the potential for expression of the NHBA peptide due to the frameshift mutations or premature stop codon. A total of 28 unique NHBA peptides were found, and of these, 13 had not been previously assigned (S2 Table). The pairwise amino acid sequence identity was at least 76.0% among the 28 different NHBA peptides (Fig 3). Twelve newly described NHBA peptides were detected only once. Despite the observed diversity of the predicted NHBA peptides, we found seven peptides, 3 (n = 16/145, 11.0%), 18 (n = 10/145, 6.9%), 20 (n = 7/145, 4.8%), 21 (n = 30/145, 20.7%), 24 (n = 13/145, 9.0%), 115 (n = 8/145, 5.5%), and 1390 (n = 24/145, 16.6%), that accounted for 74.5% of the isolates (n = 108/145). NHBA peptide 2, included as an antigen in the 4CMenB vaccine, was found in only one (0.7%) isolate. The five most common peptides were exclusively associated with unique ccs; peptides 3 (cc32), 18 (cc213), 20 (cc162), 115 (cc213), and newly described 1390 (cc461) (Fig 4).

### Genetic variability and distribution of fHbp

**Fig 5:**
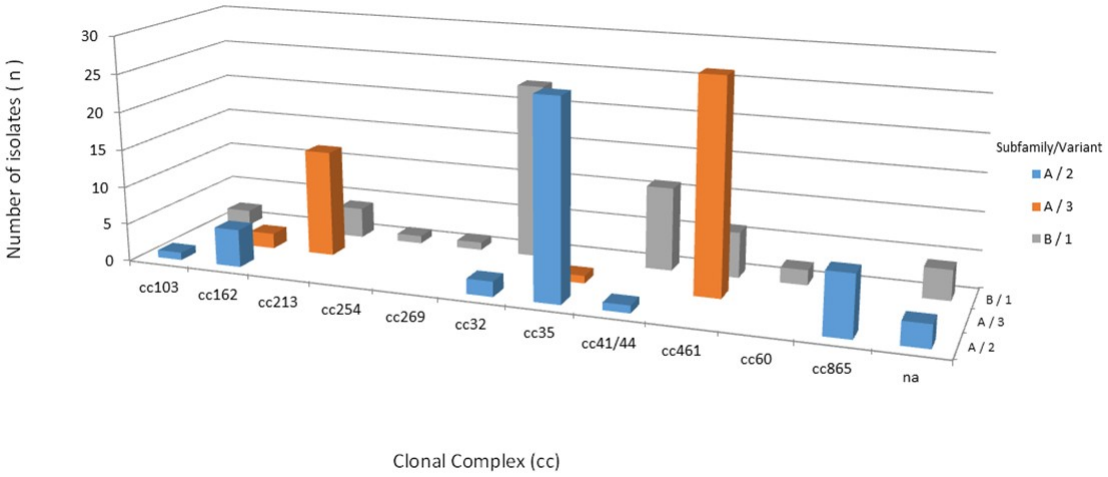
Frequency distribution of fHbp subfamily/variant among 145 serogroup B invasive meningococcal disease isolates, Brazil, 2016–2018 by clonal complex.

**Fig 6:**
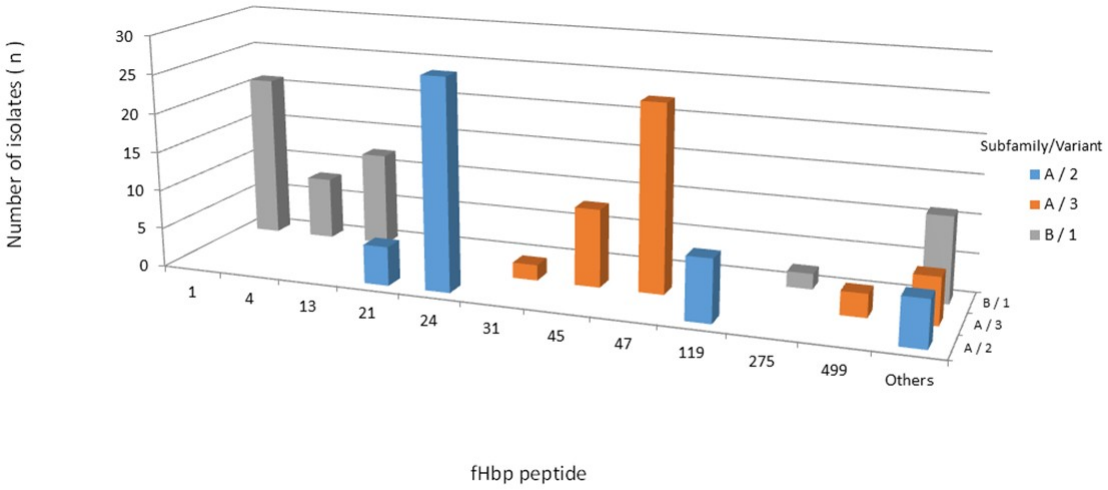
Frequency distribution of fHbp peptides among 145 serogroup B invasive meningococcal disease isolates, Brazil, 2016–2018. Others: fHbp peptides represented by only one isolate.

All 145 isolates contained the *fHbp* gene. A total of 34 unique fHbp peptides were identified: 15 belonging to subB/var1, 9 to subA/var2, and 10 to subA/var3. A total of 23 (67,6%) fHbp peptides were detected only once. Six fHbp peptides have not been previously assigned (S2 Table). A total of 54 (37.3%) isolates were predicted to encode the fHbp peptide of subB/var1, 46 (31.7%) of subA/var2 and 45 (31.0%) of subA/var3, clustering in 12 ccs. Most subB/var1 alleles belong to cc32 (n = 23/54, 42.6%), subA/var2 to cc35 (n = 26/46, 56.5%) and subA/var3 to cc461 (n = 28/45, 62.2%) (Fig 5). Moreover, we found only 12 alleles which were present in more than one isolate, despite the observed diversity of the predicted fHbp peptides (n = 35). The most prevalent fHbp peptides were 24 (subA/var2), 47 (subA/var3), 1 (subB/var1; the 4CMenB vaccine antigen), and 45 (subA/var3), accounting for 18.6% (27/145), 16.5% (24/145), 14.5% (21/145), and 6.9% (10/145) of the isolates, respectively (Fig 6). The maximum pairwise amino acid sequence identity between fHbp subA/var2-3 and subB/var1 was 61.0%. Additionally, the pairwise amino acid identity within fHbp subA/var2-3 and subB/var1 was least 87.0% and 92.0%, respectively (Fig 3).

### Genetic variability and distribution of antimicrobial resistance-associated genes

All isolates were found to present the *gyrA* gene without mutations. A total of seven *gyrA* alleles were identified; of these, only one (allele 314) had not been previously assigned (S2 Table). The *gyrA* alleles 2 (n = 57/145; 39.3%), 3 (n = 21/145; 14.5%) and 4 (n = 58/145; 40.0%) accounted for 93.8% (n = 136/145) of the isolates distributed in almost all ccs, except cc254 and cc269.

A total of 17 *rpoB* alleles were found among the 145 isolates. Five *rpoB* alleles had not been previously assigned, and four of them were found only once. The four most common *rpoB* alleles (n = 100/145, 69.0%) were almost exclusively associated with a unique cc: alleles 2, 5, 34, and 72 were related to cc32, cc461, cc213, and cc35, respectively (S2 Table).

**Fig 7:**
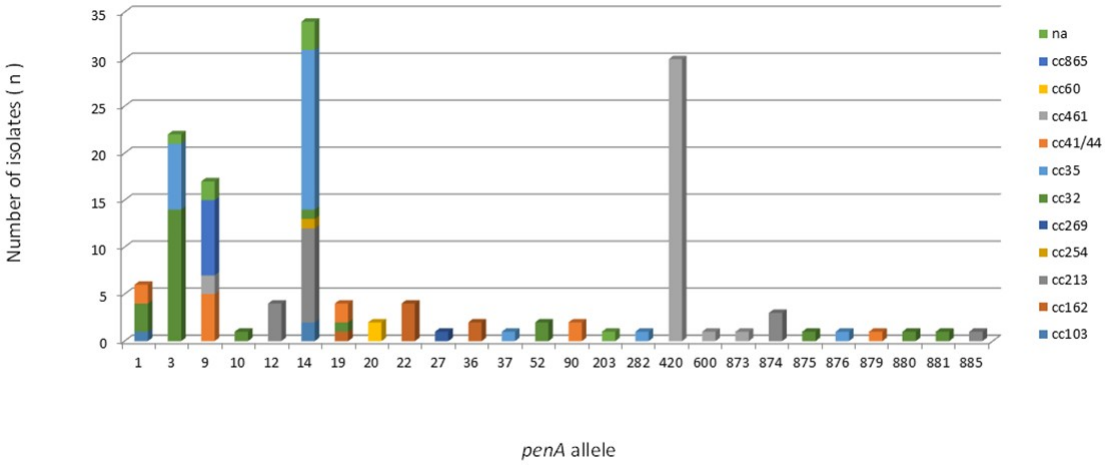
Frequency distribution of *penA* alleles among 145 serogroup B invasive meningococcal disease isolates, Brazil, 2016–2018, by clonal complex.

A total of 26 *penA* alleles were identified among the 145 isolates. Half of the alleles identified were found only once. In addition, 82/145 (56.5%) isolates carried *penA* alleles (9, 10, 12, 14, 19, 20, 36, 37, 52, 90, 203, 282, 600, 873, 874, 875, 876, 879, 880, 881 and 995) harboring reduced susceptibility-associated mutations (amino acid substitutions: F504L, A510V, I515V, G541N, and I566V). The mutated alleles were observed among all described ccs, except cc269. The nonmutated alleles 22, 27 and 420 were exclusively associated with cc162 (n = 4), cc269 (n = 1) and cc461 (n = 30), respectively. The most prevalent *penA* alleles were 14, 420, 3, and 9, which accounted for 71.0% (n = 103/145) of the isolates (Fig 7). The remaining isolates represented 4% or less. Eight mutated alleles had not been previously assigned (S2 Table).

## Discussion

Here, we present the detailed genomic analysis of a collection of MenB invasive strains from Brazil, focusing on vaccine antigens, antimicrobial resistance genes, and hyperinvasive lineage distribution. After three decades of the predominance of cc32 in the MenB clonal structure in Brazil [13, 33], we observed the important circulation of cc213, cc35, and cc461, indicating that an epidemiological change has occurred.

Our data show that there is no prevalent circulation of a single MenB cc in Brazil, which has currently been observed for serogroup B IMD epidemiology in some European countries and not in North America [15, 17, 34]. cc213, cc35, and cc461 have previously been reported in Argentina, Australia, Colombia, Paraguay, Tunisia, Turkey, and Europe [23, 22, 35–41]. Additionally, clonal diversity exhibiting a decline in cc41/44 and cc32 and an increase in cc269 and cc461 was observed in the Republic of Ireland over 19 years [39]. Variations over time that led to a change in the distribution of cc were also reported in western Australia, England, Wales, and northern Spain [14, 37, 39, 42, 43]. It is well-documented in meningococcal epidemiology that certain meningococcal lineages are known to transmit globally [44], and this may be a possible explanation for the increase in cc213, cc35, and cc461 in Brazil. Thus, further studies are needed to better understand the emergence of these clones in Brazil and how they can impact transmission in the near future.

Previous studies have demonstrated the strong and persistent association of surface antigen variants with ccs over time [45–47]. The genetic characterization among MenB strains isolated from Brazil in 2004 and among additional strains from São Paulo from 1988, 1996, and 2006 revealed that the meningococcal strain B:4,7:P1.19,15 cc32 predominated throughout Brazil [13]. The outer membrane protein (OMP) type was significantly associated with the cc. Among cc32 strains, PorB 4, 7, 11, 9, 5; PorA 19, 15, 36; and F5-1 prevailed across Brazil, except in the North Region, where F5-13 was the most prevalent [13]. In this study, we reinforced the strong association of the cc32 strain carrying PorB 3–1 (21/25, 84,5%), PorA 19 and 15 (21/25, 84,5%) and F5-1 (18/25, 72,0%). We did not detect F5-13, but only one strain from the North Region was available.

Additionally, the predominant circulation of serogroup B strains carrying fHbp peptide 1 for decades in Brazil has been described [13, 22], and a strong association between fHbp subB/var1 and peptide 1 within cc32 is well described in the literature [48]. We have shown the circulation of fHbp subB/A (var1/2–3) in almost equal proportions in recent years, in contrast to reports in China, where subA/var2 is prevalent [49], and in the United States and some European countries, where subB/var1 has prevailed among serogroup B IMD strains [50–52]. The epidemiology of fHbp subfamilies/variants has been related to different age groups. IMD cases caused by strains with fHbp subA/var2-3 have caused more disease in infants (< 1 year) than adolescents and young adults [53, 54]. Our data reinforce the previous observation; 80.5% (*p = 0*,*005*) of IMD cases in patients less than 1 year of age were due to fHbp subA/var2-3 strains. Strains with fHbp peptides 24 (subA/var2) and 47 (subA/var3) were frequent causes of IMD cases in infants <1 year of age.

Additionally, our data on *fHbp* gene variability can be explained by the emergence and expansion of new ccs in Brazil. For example, strains belonging to cc213 harbor fHbp subA/var3 (77.8%), cc35 harbors fHbp subA/var2 (96.3%), cc461 harbors fHbp subA/var3 (82.3%), and cc32 harbors fHbp subB/var1 (92.0%). In this study, we found 35 different fHbp peptides among all subfamilies/variants. Despite a high diversity, we have shown the prevalence of fHbp peptides 1, 24, 45, and 47 among the studied strains, which were predominantly associated with cc32, cc35, cc213, and cc461, respectively, as previously reported [36, 40, 41, 51].

Regarding the *nadA* gene, only 26.2% (38/145) of the studied strains presented the potential for expression of a functional peptide; this low percentage has also been reported by others, including in a study on a collection of Brazilian MenB strains [13, 43, 51]. The majority (27/38, 71.0%) of these strains had the potential of expressing NadA peptide and were also predicted to encode the fHbp subB/var1 peptide. Only 11 strains harbored fHbp subA/var2/3 in the presence of the NadA peptide. Such an observation of a close correlation of NadA and fHbp subB/var1 peptide within cc32 has also been described previously, including among Brazilian meningococcal strains [13, 43, 47, 52, 56]. cc32 was the only lineage-clustering isolate (25/38, 66.0%) presenting the potential for expression of NadA peptide, as prevalent peptide 1, as previously reported [36, 47, 55, 56]. All strains belonging to cc213 were assigned to variant 4/5, peptide 3 or 79, but we found only four frameshift peptides, an event frequently reported for cc213 [16, 36, 43].

We also detected high heterogeneity in NHBA peptides, but these prevalent NHBA peptides have been widely reported; an exception is NHBA peptide 1390, which is first described in this study. We observed a close association with types of NHBA peptides within ccs as well as those already described [36, 41, 51, 57]. In our study, cc213 includes strains that also present NHBA peptide 115, which was not observed in Spain [36]. Interestingly, we showed for the first time the association of cc461 with NHBA peptide 1390.

Considering the vaccine antigen components of the 4CMenB vaccine, fHbp peptide 1 (sub/var1) was present in 21 strains (14.5%), NHBA peptide 2 was present in 1 strain (0.7%), PorAVR2-4 was present in 15 strains (10.3%), and no strain presented NadA peptide 8. On the other hand, 10 strains (6.9%) carried fHbp peptide 45 (subA/var3), matching with one of the antigens of the MenB-FHbp vaccine. The genetic characterization of vaccine antigens is not sufficient to predict the strain coverage. In an attempt to fill this gap, two methods (MATS and MEASURE) were developed to measure the level of antigenic expression and provide predicted strain coverage by 4CMenB and MenB-FHbp vaccines, respectively [50, 58]. Recently, a genetic MATS (gMATS) was proposed for predicting strain coverage by an association of antigen genotyping and MATS results [59].

Temporal changes in the prevalence of vaccine antigen variants can impact predicted MenB strain coverage. Recently, a decrease in the potential predicted coverage of MenB strains by the 4CMenB vaccine was observed in England, Wales, Northern Ireland, North Spain, and The Netherlands [37, 39, 42]. MATS was performed in a Brazilian collection of 99 serogroup B IMD strains isolated in 2010, and the strain coverage predicted by MATS was 80.8%. There is no information about fHbp, NHBA, or NadA peptides in this studied collection. However, we can assume that the predicted coverage of MenB strains can be attributed to the predominance of cc32 mostly associated with fHbp peptide 1 (subB/v1) and to the prevalence of NHBA peptide cross-reactivity with the 4CMenB vaccine [60].

Half of the studied MenB strains belonged to three antigenic distinct emerging ccs: cc461, 35, and 213. Therefore, we identified a shift in the prevalent repertoire of the antigens of the serogroup B IMD strains. The predominance of fHbp subB/var1 decreased between 2004 (86.7%) and 2016–2018 (31.7%) due to circulation of cc461, cc35, and 213, which predominantly harbored fHbp subA/var2–3. It was observed that fHbp subA/var2-3 or subB/var1 antigens elicit mainly subfamily specific responses and that these are cross-protective against strains expressing variants from the same subfamily [58].

The emergence of these ccs carrying completely unknown peptides makes it difficult to estimate the real vaccine predicted coverage, since no experimental data are available. Most strains belonging to cc461 harbored fHbp peptide 47 (subA/var3), which is not covered by gMATS and had NHBA peptide 1390, which is unpredictable by gMATS or MATS (S2 Table) [59]. Most strains belonging to cc35 harbored fHbp peptide 24 (subA/var2), which is not covered by gMATS but did have NHBA peptide 21, which is covered by gMATS (S2 Table) [59]. All strains belonging to cc461, and cc35 did not have NadA or PorAVR2-4. Most strains belonging to cc213 harbor fHbp peptide 45 (subA/var3) and NHBA peptide 18, which are not covered by gMATS, or had NHBA peptide 115, which is unpredictable by gMATS (S2 Table) [59]. All strains belonging to cc213 had NadA 4/5, which has no cross-reactivity between NadA variant 2/3 [56]. On the other hand, the fHbp peptides 47 and 45 are indicated as being potentially susceptible to the MenB-FHbp vaccine, and there is insufficient data on fHbp peptide 24 to predict coverage by this vaccine (S2 Table) [61]. In light of the large antigenic variability found in this study, an ongoing investigation will provide more contribution to the cross-reactivity of this studied strain collection with available MenB vaccines based on genomic data. However, our data will be limited by the lack of cross-reactivity data from the new peptides with the available vaccines.

Although meningococcal resistance to ciprofloxacin remains uncommon, strains not susceptible to the drug have been reported sporadically in other countries, including Brazil [62–65]. Our results showed that Men strains remain extremely susceptible to ciprofloxacin, and we did not find a mutated *gyrA* allele. Currently, rifampicin is one of the recommended antimicrobials for chemoprophylaxis for IMD in Brazil [65]. Previous studies have already shown rifampicin resistance [66]. However, no mutated *rpoB* gene was characterized in our study. Therefore, our data strengthen the indication for the use of rifampicin as a drug that can be used for IMD chemoprophylaxis in Brazil.

Alterations in the *penA* gene encoding penicillin-binding protein 2 (PBP2) seem to be the most common mechanism of penicillin resistance [67]. Our findings indicate that the frequency of reduced susceptibility to penicillin remains high in Brazil. An upward trend of reduced susceptibility to penicillin has also been reported in other countries [68, 69]. Interestingly, we observed the exclusive association of nonmutated *penA* allele 420 with cc461, which is in contrast to what was observed in Tunisia, where cc461 is associated with the mutated *penA* allele 33 [40]. Our data suggest a clonal expansion of cc461 associated with reduced susceptibility to penicillin. An association of serotype 19 within Brazilian serogroup B strains with this type of resistance has been previously described [70]. Other mechanisms for inducing reduced susceptibility to penicillin without mutation of the *penA* gene have been described [71]. Overall, more research is required to determine which type of mechanism is involved in cc461.

Important limitations of this study should be considered. The quality of epidemiological surveillance and meningococcal diagnosis differ among various geographic regions of the country. Although there has been an improvement with the incorporation of qPCR in our surveillance system, approximately one-third of IMD cases are still confirmed by clinical criteria, and only 47% of the IMD cases reported during the study period had serogroup information [2]. Therefore, we might have underestimated the real burden of IMD cases in Brazil. Nonetheless, we are aware that in the era of the dramatic spread of the COVID-19 pandemic in Latin America, continuous surveillance of IMD cases is key in monitoring the emergence of possible cases of IMD secondary to COVID-19, as recently observed in France [72].

A considerable amount of additional work will be needed to describe the antigenic diversity of the *N*. *meningitidis* strains circulating in Latin America. However, this study makes several noteworthy contributions to understanding the temporal changes in vaccine antigens as well as knowledge of emerging hyperinvasive lineages of *N*. *meningitidis* strains circulating in our region. The present data reinforce that continued laboratory surveillance over time supports local epidemiological data as the driver in planning and preparedness in making decisions about the best vaccination schedule strategy against serogroup B IMD.

## Supporting information

S1 Fig Incidence rates of invasive meningococcal disease by serogroup: Brazil, 2000–2018. (TIF)

S2 Fig Incidence rates of serogroup B invasive meningococcal disease caused by age group: Brazil, 2000–2018. (TIF)

S3 Fig Circular tree representation based on high-quality SNPs (CSIPhylogeny) of 145 serogroup B invasive meningococcal disease Brazilian isolates. Colored circles represent the ST based on the seven housekeeping genes (as determined at neisseria.org (https://pubmlst.org/organisms/neisseria-spp/). The rings (from the inner) represent the following: 1. presence of NadA peptide; 2. fHbp peptide (subfamily/variant); 3. most frequent NHBA peptides found in this study (115, 1390, 18, 20, 3, and the remaining ones grouped as ‘other’); 4. most frequent PorA variants; 5. most frequent PorB variants; and 6. most frequent FetA variants.
(TIF)

S1 Table Epidemiological data for 145 studied *Neisseria meningitidis* serogroup B invasive isolates from Brazil, 2016–2018. (XLSX)

S2 Table Genomic characterization of serogroup B invasive meningococcal disease strains (n = 145) isolated in Brazil from 2016 to 2018. (DOCX)
