# Supplementary figures and images for: Genomic surveillance of Neisseria meningitidis serogroup B invasive strains: Diversity of vaccine antigen types, Brazil, 2016-2018
Source: PLoS One. 2020 Dec 21;15(12):e0243375. doi: 10.1371/journal.pone.0243375 (PMC7751880; doi:10.1371/journal.pone.0243375)

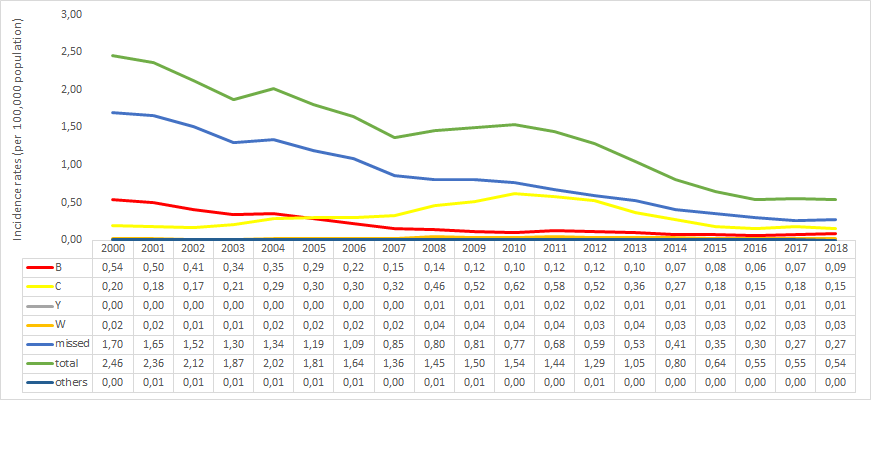

Supplement: S1 Fig — (TIF) [file pone.0243375.s001.tif]

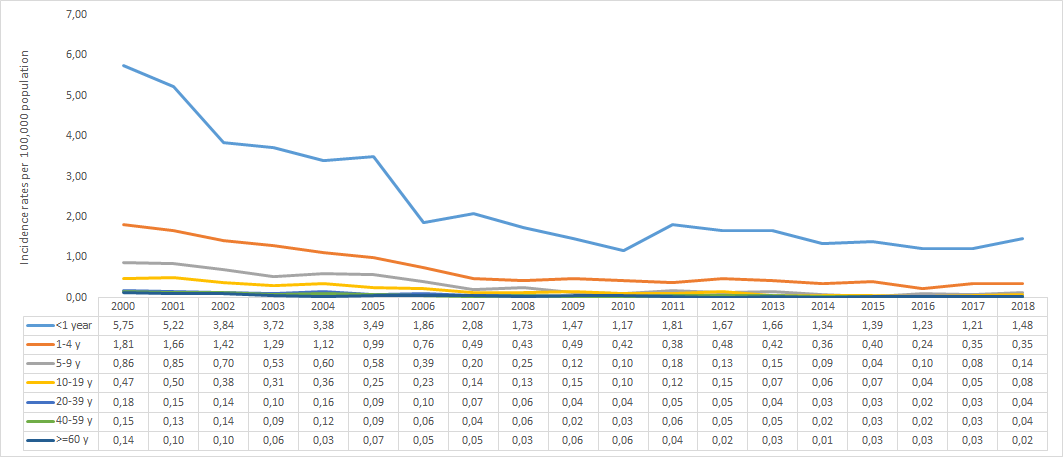

Supplement: S2 Fig — (TIF) [file pone.0243375.s002.tif]

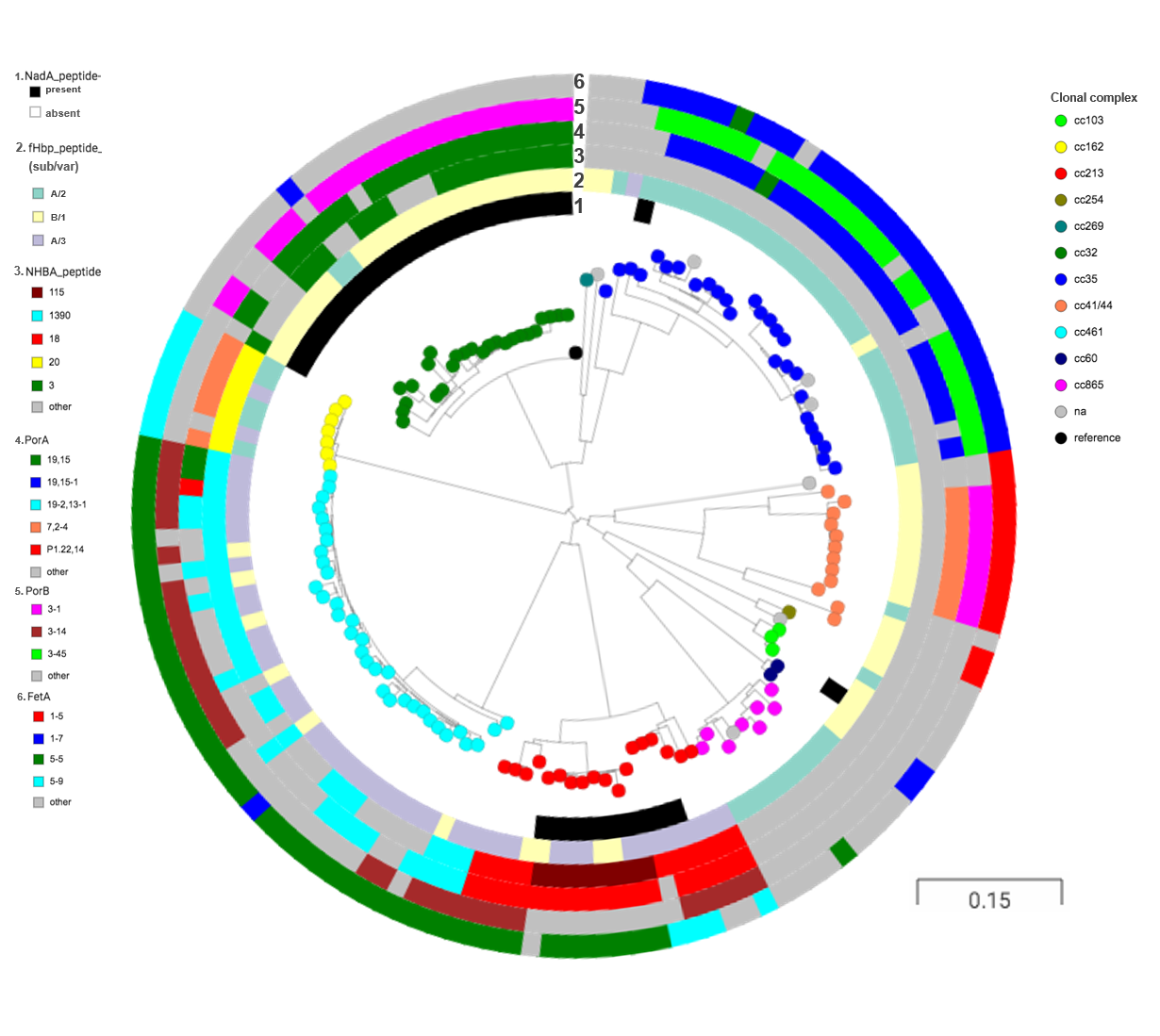

Supplement: S3 Fig — The rings (from the inner) represent the following: 1. presence of NadA peptide; 2. fHbp peptide (subfamily/variant); 3. most frequent NHBA peptides found in this study (115, 1390, 18, 20, 3, and the remaining ones grouped as ‘other’); 4. most frequent PorA variants; 5. most frequent PorB variants; and 6. most frequent FetA variants. (TIF) [file pone.0243375.s003.tif]
