# Supplementary material for: Genomic surveillance of Neisseria meningitidis serogroup B invasive strains: Diversity of vaccine antigen types, Brazil, 2016-2018
Source: PLoS One. 2020 Dec 21;15(12):e0243375. doi: 10.1371/journal.pone.0243375 (PMC7751880; doi:10.1371/journal.pone.0243375)
Supplement: S2 Table — (DOCX) [file pone.0243375.s005.docx]

**S2 Table**. Genomic characterization of serogroup B invasive meningococcal disease strains (n=145) isolated in Brazil from 2016 to 2018.

| **No. of strain** | **Acession Number GenBank** | **ST** | **Clonal Complex** | **fHbp peptide**  **(reactivity for 4CMenB vaccine)** | **fHbp peptide**  **(reactivity for**  **Men-FHbp vaccine)** | **fHbp**  **variant** | **fHbp**  **subfamily** | **NadA peptide**  **(reactivity for 4CMenB vaccine)** | **NadA**  **variant** | ***nhba***  **allele** | **NHBA Peptide**  **(reactivity for 4CMenB vaccine)** | **PorB** | **PorA VR1** | **PorA VR2**  **(reactivity for 4CMenB vaccine)** | **FetAVR** | ***penA*** | ***gyrA*** | ***rpoB*** |
| --- | --- | --- | --- | --- | --- | --- | --- | --- | --- | --- | --- | --- | --- | --- | --- | --- | --- | --- |
| N.26/16 | SJTI00000000 | 461 | cc461 | 47 | 47 | 3 | A | 0 | 0 | **1562** | **1390** | 3-14 | 19-2 | 13 | 5-5 | 420 | 2 | 5 |
| N.31/16 | SJTM00000000 | **14742** | cc254 | 13 | 13 | 1 | B | 0 | 0 | 7 | 9 | **3-1011** | 5-1 | 10-13 | 3-9 | 14 | 11 | 42 |
| N.44/16 | SEUY00000000 | 213 | cc213 | 499 | 499 | 3 | A | 0 | NadA-4/5 | 33 | 18 | 3-14 | 22 | 14 | 5-5 | **874** | 3 | 34 |
| N.48/16 | SEUZ00000000 | 213 | cc213 | 275 | 275 | 1 | B | 122 | NadA-4/5 | 258 | 115 | 3-350 | 22 | 14 | 5-5 | 14 | 3 | 34 |
| N.55/16 | SWJP00000000 | 485 | cc41/44 | 4 | 4 | 1 | B | 0 | 0 | 65 | 58 | 3-1 | 7-2 | 4 | 1-5 | 9 | 4 | 18 |
| N.57/16 | SEUO00000000 | 461 | cc461 | 13 | 13 | 1 | B | 0 | 0 | **1608** | **1434** | 3-14 | 19-2 | 13-15 | 5-5 | 420 | 2 | 5 |
| N.70/16 | SJTP00000000 | 11827 | cc35 | 24 | 24 | 2 | A | 0 | 0 | 19 | 21 | 3-45 | 19 | 15-1 | 1-7 | 14 | 4 | 72 |
| N.78/16 | SJTU00000000 | 461 | cc461 | **1233** | **1233** | 1 | B | 0 | 0 | **1562** | **1390** | 3-14 | 19-2 | 13-1 | 5-5 | 600 | 2 | 5 |
| N.82/16 | SEUR00000000 | 213 | cc213 | 45 | 45 | 3 | A | 122 | NadA-4/5 | 258 | 115 | 3-350 | 22 | 14 | 5-5 | 14 | 3 | 34 |
| N.84/16 | SJTJ00000000 | 3496 | cc213 | 45 | 45 | 3 | A | 0 | 0 | 33 | 18 | 3-14 | 22 | 14 | 3-6 | 14 | 3 | 28 |
| N.85/16 | SPLZ00000000 | 3764 | cc32 | 1 | 1 | 1 | B | 118 | NadA-1 | **1747** | **1560** | 3-79 | 7-1 | 1-4 | 5-1 | 19 | 2 | 2 |
| N.86/16 | SEUQ00000000 | 11827 | cc35 | 24 | 24 | 2 | A | 0 | 0 | 19 | 21 | 3-45 | 19 | 15-1 | 1-7 | 37 | 4 | **263** |
| N.89/16 | SEUW00000000 | 213 | cc213 | 499 | 499 | 3 | A | 0 | NadA-4/5 | 33 | 18 | 3-14 | 22 | 14 | 5-5 | **874** | 3 | 34 |
| N.92/16 | SJTW00000000 | 461 | cc461 | 47 | 47 | 3 | A | 0 | 0 | **1562** | **1390** | 3-14 | 19 | 15 | 5-5 | 420 | 2 | 5 |
| N.96/16 | SEUS00000000 | 34 | cc32 | 1 | 1 | 1 | B | 1 | NadA-1 | 334 | 321 | 3-1 | 19 | 15 | 5-1 | **875** | 2 | 2 |
| N.97/16 | SEUT00000000 | 3327 | cc865 | 119 | 119 | 2 | A | 0 | 0 | 15 | 24 | 3-106 | 21 | 16-36 | 5-5 | 9 | 4 | 40 |
| N.105/16 | SPLN00000000 | 485 | cc41/44 | 4 | 4 | 1 | B | 0 | 0 | 65 | 58 | 3-1 | 7-2 | 4 | 1-5 | 9 | 4 | 18 |
| N.106/16 | SJTO00000000 | 485 | cc41/44 | 4 | 4 | 1 | B | 0 | 0 | 65 | 58 | 3-1 | 7-2 | 4 | 1-5 | 9 | 4 | 18 |
| N.113/16 | SEUP00000000 | 3085 | cc35 | 552 | 552 | 3 | A | 0 | 0 | 19 | 21 | 3-84 | 7-2 | 13 | 1-7 | **876** | 4 | 72 |
| N.118/16 | SJTN00000000 | 3327 | cc865 | 119 | 119 | 2 | A | 0 | 0 | 15 | 24 | 3-106 | 21 | 16-36 | 5-8 | 9 | 4 | 40 |
| N.128/16 | VHNV00000000 | 3496 | cc213 | 45 | 45 | 3 | A | 0 | 0 | 33 | 18 | 3-14 | 22 | 14 | 5-2 | 12 | 3 | 34 |
| N.136/16 | SPMS00000000 | **14743** | cc32 | 1 | 1 | 1 | B | 1 | NadA-1 | 5 | 3 | 3-1 | 18-1 | 30-3 | 5-1 | 3 | 2 | 2 |
| N.143/16 | SEUU00000000 | **14744** | cc461 | 47 | 47 | 3 | A | 0 | 0 | **1562** | **1390** | 3-14 | 18 | 25-15 | 5-5 | 420 | 2 | 5 |
| N.151/16 | SPMU00000000 | 461 | cc461 | 47 | 47 | 3 | A | 0 | 0 | **1562** | **1390** | **3-986** | 19-2 | 13-1 | 5-5 | 420 | 2 | 5 |
| N.161/16 | SEUV00000000 | **14626** | cc461 | 47 | 47 | 3 | A | 0 | 0 | **1562** | **1390** | 3-14 | 19-2 | 13 | 5-5 | 420 | 2 | 5 |
| N.166/16 | SEUX00000000 | **14629** | cc865 | 346 | 346 | 2 | A | 0 | 0 | 15 | 24 | 3-106 | 21 | 16-36 | 5-8 | 9 | 4 | 40 |
| N.170/16 | SJTK00000000 | 461 | cc461 | 45 | 45 | 3 | A | 0 | 0 | **1562** | **1390** | 3-14 | 19-2 | 13-1 | 5-5 | 420 | 2 | 5 |
| N.171/16 | SJTL00000000 | 162 | cc162 | 21 | 21 | 2 | A | 0 | 0 | 11 | 20 | 3-631 | 7-2 | 4 | 5-9 | 19 | 4 | 28 |
| N.179/16 | SWJN00000000 | 162 | cc162 | 21 | 21 | 2 | A | 0 | 0 | 11 | 20 | 3-81 | 7-2 | 4 | 5-9 | 22 | 4 | 28 |
| N.181/16 | SPLY000000000 | 269 | cc269 | 15 | 15 | 1 | B | 0 | 0 | 14 | 21 | 3-25 | 19-1 | 15-11 | 5-1 | 27 | 12 | 4 |
| N.182/16 | SPMV00000000 | **14630** | na | 119 | 119 | 2 | A | 0 | 0 | 1305 | 24 | 3-106 | 7 | 16-36 | 5-8 | 9 | 4 | 40 |
| N.187/16 | SJTT00000000 | 11827 | cc35 | 24 | 24 | 2 | A | 0 | 0 | 19 | 21 | 3-45 | 19 | 0 | 1-7 | 14 | 4 | 72 |
| N.200/16 | SJTV00000000 | 11827 | cc35 | 24 | 24 | 2 | A | 0 | 0 | 19 | 21 | 3-45 | 19 | 15-1 | 1-7 | 3 | 4 | 72 |
| N.213/16 | SJTX00000000 | **14574** | cc41/44 | 14 | 14 | 1 | B | 0 | 0 | 1 | 2 | 3-64 | 7-2 | 13-9 | 1-5 | 1 | 4 | 18 |
| N.225/16 | SJTY00000000 | 437 | cc41/44 | 19 | 19 | 2 | A | 0 | 0 | 37 | 1 | 3-671 | 22-1 | 14 | 5-2 | 1 | 2 | 18 |
| N.227/16 | SJTZ00000000 | **14575** | cc32 | 119 | 119 | 2 | A | 1 | NadA-1 | 5 | 3 | 3-1 | 19 | 15 | 5-1 | 1 | 2 | 2 |
| N.228/16 | VKJY00000000 | 33 | cc32 | 1 | 1 | 1 | B | 1 | NadA-1 | 5 | 3 | 3-1 | 19 | 15 | 1-68 | 3 | 2 | 2 |
| N.245/16 | SJUN00000000 | 461 | cc461 | 47 | 47 | 3 | A | 0 | 0 | **1562** | **1390** | 3-14 | 19-2 | 13-1 | 5-5 | 420 | 2 | 5 |
| N.246/16 | SJUA00000000 | 3780 | cc103 | 25 | 25 | 2 | A | 0 | 0 | 15 | 24 | 2-23 | 22 | 14-6 | 3-9 | 22 | 3 | 6 |
| N.248/16 | SJUB00000000 | 162 | cc162 | 21 | 21 | 2 | A | 0 | 0 | 11 | 20 | 3-81 | 7-2 | 4 | 5-9 | 36 | 4 | 28 |
| N.249/16 | SWJI00000000 | 213 | cc213 | 499 | 499 | 3 | A | 0 | NadA-4/5 | 33 | 18 | 3-14 | 22 | 14 | 5-5 | **874** | 3 | 34 |
| N.251/16 | SJUC00000000 | 213 | cc213 | 174 | 174 | 3 | A | 122 | NadA-4/5 | 258 | 115 | 3-350 | 22 | 14 | 5-5 | 14 | 3 | 34 |
| N.253/16 | SJUD00000000 | 11827 | cc35 | 24 | 24 | 2 | A | 0 | 0 | 19 | 21 | **3-1014** | 19 | 15-1 | 1-7 | 14 | 4 | 72 |
| N.254/16 | SJUE00000000 | 33 | cc32 | 1 | 1 | 1 | B | 1 | NadA-1 | 5 | 3 | 3-1 | 19 | 15 | 1-68 | 3 | 2 | 2 |
| N.256/16 | SPML00000000 | 461 | cc461 | 1220 | 1220 | 1 | B | 0 | 0 | **1562** | **1390** | 3-14 | 19-2 | 13-9 | 5-5 | 420 | 2 | 5 |
| N.262/16 | VKJX00000000 | 3496 | cc213 | 45 | 45 | 3 | A | 79 | NadA-4/5 | 33 | 18 | 3-64 | 22 | 14-24 | 5-9 | 12 | 3 | 34 |
| N.263/16 | SPMM00000000 | 35 | cc35 | 16 | 16 | 2 | A | 0 | 0 | 19 | 21 | 3-39 | 22-1 | 14 | 1-1 | 282 | 4 | 31 |
| N.275/16 | SJUF00000000 | 11827 | cc35 | 24 | 24 | 2 | A | 0 | 0 | 19 | 21 | **3-1016** | 7 | 30-3 | 1-7 | 14 | 4 | 72 |
| N.1/17 | SPMA00000000 | 461 | cc461 | 47 | 47 | 3 | A | 0 | 0 | **1562** | **1390** | 3-14 | 19-2 | 13-1 | 5-5 | 420 | 2 | 5 |
| N.2/17 | SPMB00000000 | 3327 | cc865 | **161** | **161** | 2 | A | 0 | 0 | 15 | 24 | **3-985** | 21 | 16-36 | 5-8 | 9 | 4 | 40 |
| N.3/17 | SPMC00000000 | 461 | cc461 | 47 | 47 | 3 | A | 0 | 0 | **1562** | **1390** | 3-14 | 7-1 | 1 | 5-5 | 420 | 2 | 5 |
| N.14/17 | SPMD00000000 | 12020 | cc103 | 13 | 13 | 1 | B | 0 | 0 | 15 | 24 | 2-23 | 22 | 14-6 | 3-9 | 14 | 4 | 6 |
| N.23/17 | SPME00000000 | 461 | cc461 | 47 | 47 | 3 | A | 0 | 0 | 92 | 118 | 3-35 | 19-2 | 13-1 | 5-5 | **873** | 2 | 5 |
| N.25/17 | SPMF00000000 | 461 | cc461 | 47 | 47 | 3 | A | 0 | 0 | **1562** | **1390** | 3-14 | 22 | 14 | 5-5 | 420 | 2 | 5 |
| N.27/17 | SPMG00000000 | 461 | cc461 | 31 | 31 | 3 | A | 0 | 0 | 92 | 118 | 3-41 | 19-2 | 13-1 | 5-5 | 420 | 2 | 5 |
| N.28/17 | SPMH00000000 | 639 | cc32 | 1221 | 1221 | 1 | B | 1 | NadA-1 | **1745** | **1559** | 3-1 | 19 | 15 | 5-1 | 1 | 2 | 2 |
| N.31/17 | SWJH00000000 | **15545** | na | 24 | 24 | 2 | A | 0 | 0 | 19 | 21 | 3-45 | 19 | 15-1 | 1-7 | 3 | 4 | 72 |
| N.41/17 | SPLS00000000 | **14577** | na | 24 | 24 | 2 | A | 0 | 0 | **1606** | **1433** | 3-45 | 19 | 15-1 | 1-7 | 14 | 4 | 72 |
| N.42/17 | SPLT00000000 | 34 | cc32 | 1 | 1 | 1 | B | 1 | NadA-1 | 5 | 3 | 3-1 | 19 | 15 | 5-1 | 3 | 2 | 2 |
| N.43/17 | SPSI00000000 | 485 | cc41/44 | 4 | 4 | 1 | B | 0 | 0 | 65 | 58 | 3-1 | 7-2 | 4 | 1-5 | 9 | 4 | 18 |
| N.46/17 | SPLQ00000000 | 461 | cc461 | 47 | 47 | 3 | A | 0 | 0 | **1562** | **1390** | 3-14 | 19-2 | 13-1 | 5-5 | 9 | 2 | 5 |
| N.60/17 | SPLR00000000 | 3327 | cc865 | 119 | 119 | 2 | A | 0 | 0 | **1609** | **1435** | 3-106 | 21-7 | 16-36 | 4-28 | 9 | 4 | 40 |
| N.63/17 | SPMI00000000 | **14635** | cc461 | 47 | 47 | 3 | A | 0 | 0 | **1562** | **1390** | **3-1190** | 19-2 | 13-1 | 5-5 | 420 | 2 | 5 |
| N.64/17 | SPLU00000000 | 461 | cc461 | 47 | 47 | 3 | A | 0 | 0 | **1562** | **1390** | 3-35 | 19-2 | 13 | 5-5 | 420 | 2 | **258** |
| N.91/17 | SPLV00000000 | **14578** | na | 13 | 13 | 1 | B | 0 | 0 | 19 | 21 | **3-1013** | 19 | 15-1 | 1-7 | 14 | 4 | 72 |
| N.93/17 | VHNK00000000 | 12020 | cc103 | 13 | 13 | 1 | B | 118 | NadA-1 | 15 | 24 | 2-23 | 22 | 14-6 | 3-9 | 14 | 4 | 6 |
| N.96/17 | SPLW00000000 | 213 | cc213 | 45 | 45 | 3 | A | 122 | NadA-4/5 | 258 | 115 | **3-1010** | 22 | 14 | 5-5 | 14 | 3 | 34 |
| N.97/17 | SJTQ00000000 | 3496 | cc213 | 45 | 45 | 3 | A | 79 | NadA-4/5 | 33 | 18 | 3-14 | 22 | 14 | 5-9 | 12 | 3 | 34 |
| N.115/17 | SJTR00000000 | 461 | cc461 | 47 | 47 | 3 | A | 0 | 0 | **1742** | **1557** | **3-986** | 19-2 | 13-2 | 5-5 | 420 | 2 | 5 |
| N.118/17 | SJTS00000000 | 11827 | cc35 | 24 | 24 | 2 | A | 0 | 0 | 19 | 21 | 3-45 | 19 | 15-1 | 1-7 | 14 | 4 | 72 |
| N.122/17 | SJUJ00000000 | 11403 | cc32 | 226 | 226 | 1 | B | 118 | NadA-1 | **1743** | **1558** | 3-79 | 7-1 | 1 | 1-25 | **880** | 2 | 2 |
| N.128/17 | SJUK00000000 | 34 | cc32 | 1 | 1 | 1 | B | 1 | NadA-1 | 5 | 3 | 3-1 | 19 | 15 | 5-1 | 3 | 2 | 2 |
| N.130/17 | SJUL00000000 | 34 | cc32 | 1 | 1 | 1 | B | 1 | NadA-1 | 5 | 3 | 3-1 | 19 | 15 | 5-1 | 3 | 2 | 2 |
| N.137/17 | SJUM00000000 | 278 | cc35 | 24 | 24 | 2 | A | 1 | NadA-1 | 19 | 21 | 3-45 | 18-7 | 9 | 1-7 | 14 | 4 | 72 |
| N.138/17 | SPMT00000000 | 11827 | cc35 | 24 | 24 | 2 | A | 0 | 0 | 19 | 21 | 3-45 | 19 | 15-1 | 1-7 | 14 | 4 | 72 |
| N.139/17 | SPLX00000000 | 162 | cc162 | 21 | 21 | 2 | A | 0 | 0 | 11 | 20 | 3-81 | 7-2 | 4 | 5-9 | 36 | 4 | 28 |
| N.150/17 | SPMJ00000000 | 213 | cc213 | 275 | 275 | 1 | B | 122 | NadA-4/5 | 258 | 115 | 3-350 | 22 | 14 | 5-5 | 14 | 3 | 34 |
| N.174/17 | SPMK00000000 | 34 | cc32 | 1 | 1 | 1 | B | 1 | NadA-1 | 5 | 3 | 3-1 | 19 | 15 | **5-194** | **881** | 2 | 2 |
| N.192/17 | VKKA00000000 | 7983 | cc60 | 13 | 13 | 1 | B | 0 | 0 | 15 | 24 | 2-65 | 5 | 2 | 1-7 | 20 | 3 | 4 |
| N.220/17 | SPSQ00000000 | 461 | cc461 | 47 | 47 | 3 | A | 0 | 0 | **1602** | **1429** | 3-38 | 19-2 | 13-2 | 5-5 | 420 | 2 | 5 |
| N.228/17 | VKJQ00000000 | 461 | cc461 | 47 | 47 | 3 | A | 0 | 0 | **1562** | **1390** | **3-986** | 19-2 | 13-7 | 5-5 | 420 | 2 | 5 |
| N.230/17 | SPMN00000000 | 639 | cc32 | 119 | 119 | 2 | A | 1 | NadA-1 | **1603** | **1430** | **3-987** | 19 | 15 | 1-7 | 1 | 2 | 2 |
| N.234/17 | SPSJ00000000 | 213 | cc213 | 45 | 45 | 3 | A | 122 | NadA-4/5 | 258 | 115 | 3-350 | 22 | 14 | 5-5 | 14 | 3 | 34 |
| N.235/17 | VHNW00000000 | 213 | cc213 | 45 | 45 | 3 | A | 122 | NadA-4/5 | 258 | 115 | 3-350 | 22 | 14 | 5-5 | 14 | 3 | 34 |
| N.239/17 | SPM000000000 | 213 | cc213 | 62 | 62 | 1 | B | 122 | NadA-4/5 | 258 | 115 | 3-350 | 22 | 14 | 1-19 | 14 | 3 | 34 |
| N.244/17 | SPMQ00000000 | 409 | cc41/44 | 13 | 13 | 1 | B | 0 | 0 | 0 | 0 | 3-82 | 18-1 | 34-4 | 1-5 | 19 | 2 | 27 |
| N.261/17 | SPMR00000000 | 749 | cc32 | 1 | 1 | 1 | B | 3 | NadA-2/3 | 211 | 120 | 3-1 | 19 | 15 | 5-1 | 52 | 2 | 2 |
| N.01/18 | RWIW00000000 | 11827 | cc35 | 24 | 24 | 2 | A | 0 | 0 | 19 | 21 | 3-45 | 19 | 15-1 | 1-7 | 14 | 4 | 72 |
| N.02/18 | RWIX00000000 | 11827 | cc35 | 24 | 24 | 2 | A | 0 | 0 | 19 | 21 | 3-45 | 19 | 15-1 | 1-7 | 3 | 4 | 72 |
| N.03/18 | RWIY00000000 | 461 | cc461 | **1234** | **1234** | 1 | B | 0 | 0 | **1562** | **1390** | 3-14 | 19-2 | 13 | 5-5 | 420 | 2 | 5 |
| N.08/18 | RWIZ00000000 | 11827 | cc35 | 24 | 24 | 2 | A | 0 | 0 | 19 | 21 | 3-45 | 19 | 15-1 | 1-7 | 14 | 4 | 72 |
| N.10/18 | RWJA00000000 | 3496 | cc213 | 187 | 187 | 3 | A | 79 | NadA-4/5 | 33 | 18 | 3-14 | 22 | 14 | 5-9 | 12 | 3 | 34 |
| N.11/18 | RWJB00000000 | 11827 | cc35 | 24 | 24 | 2 | A | 0 | 0 | 19 | 21 | **3-1015** | 19 | 15-1 | 1-7 | 3 | 4 | 72 |
| N.18/18 | RWJC00000000 | 461 | cc461 | 31 | 31 | 3 | A | 0 | 0 | 92 | 118 | 3-41 | 19-2 | 13-1 | 5-5 | 420 | 2 | 5 |
| N.30/18 | RWJD00000000 | 461 | cc461 | 47 | 47 | 3 | A | 0 | 0 | **1562** | **1390** | 3-14 | 19 | 15 | 5-5 | 420 | 2 | 5 |
| N.35/18 | RWJE00000000 | 162 | cc162 | **1231** | **1231** | 3 | A | 0 | 0 | 11 | 20 | **3-1012** | 7-2 | 4 | 5-9 | 22 | 4 | 28 |
| N.36/18 | RYYY00000000 | 3327 | cc865 | 119 | 119 | 2 | A | 0 | 0 | 15 | 24 | 3-106 | 21 | 16-36 | **4-78** | 9 | 4 | 40 |
| N.46/18 | RYYX00000000 | **14576** | na | 13 | 13 | 1 | B | 0 | 0 | 17 | 29 | **3-1011** | 21 | 16 | 3-9 | 14 | 11 | 42 |
| N.47/18 | RYYZ00000000 | 11827 | cc35 | 24 | 24 | 2 | A | 0 | 0 | 19 | 21 | 3-45 | 19 | 15-1 | 1-7 | 3 | 4 | 72 |
| N.54/18 | RYZA00000000 | 461 | cc461 | 47 | 47 | 3 | A | 0 | 0 | **1607** | 118 | 3-14 | 19-2 | 13-2 | 5-5 | 420 | 2 | 5 |
| N.62/18 | RYZB00000000 | 11827 | cc35 | 24 | 24 | 2 | A | 0 | 0 | 19 | 21 | 3-45 | 19 | 15-1 | 1-7 | 14 | 4 | 72 |
| N.63/18 | RYZC00000000 | 409 | cc41/44 | 13 | 13 | 1 | B | 0 | 0 | 0 | 0 | 3-82 | 18-1 | 34-4 | 1-5 | 19 | 2 | 27 |
| N.65/18 | RYZD00000000 | 461 | cc461 | 13 | 13 | 1 | B | 0 | 0 | **1562** | **1390** | 3-14 | 19-2 | 13 | 5-5 | 420 | 4 | 5 |
| N.66/18 | RYZE00000000 | 461 | cc461 | 13 | 13 | 1 | B | 0 | 0 | **1610** | **1390** | **3-986** | 19-2 | 13-1 | 5-5 | 420 | 2 | 5 |
| N.67/18 | RYZF00000000 | 33 | cc32 | 1 | 1 | 1 | B | 3 | NadA-2/3 | 211 | 120 | 3-1 | 19 | 15 | 5-1 | 52 | 2 | 2 |
| N.73/18 | RYZG00000000 | 3327 | cc865 | **1232** | **1232** | 2 | A | 0 | 0 | 15 | 24 | 3-106 | 21 | 16-36 | 5-8 | 9 | 4 | 40 |
| N.80/18 | RYZM00000000 | 461 | cc461 | 47 | 47 | 3 | A | 0 | 0 | **1562** | **1390** | 3-14 | 19-19 | 13 | 5-5 | 420 | 2 | 5 |
| N.83/18 | RYZN00000000 | 461 | cc461 | 178 | 178 | 3 | A | 0 | 0 | 92 | 118 | 3-14 | 19-2 | 13 | 5-5 | 420 | 2 | 5 |
| N.86/18 | RYZO00000000 | 34 | cc32 | 1 | 1 | 1 | B | 1 | NadA-1 | 5 | 3 | 3-1 | 19 | 15 | 1-68 | 14 | 2 | 2 |
| N.90/18 | SEIW00000000 | 485 | cc41/44 | 4 | 4 | 1 | B | 0 | 0 | 0 | 0 | 3-1 | 7-2 | 4 | 1-5 | **879** | 4 | 18 |
| N.92/18 | SEIX000000002 | 4221 | na | 1230 | 1230 | 1 | B | 0 | 0 | **1746** | **1561** | 3-475 | 7 | 0 | 5-148 | 203 | 12 | 42 |
| N.101/18 | SPSH00000000 | 11827 | cc35 | 24 | 24 | 2 | A | 0 | 0 | 19 | 21 | 3-45 | 19 | 15-1 | 1-80 | 3 | 4 | 72 |
| N.102/18 | SPMP00000000 | 11827 | cc35 | 24 | 24 | 2 | A | 0 | 0 | 19 | 21 | 3-45 | 19 | 15 | 1-7 | 3 | 4 | 72 |
| N.103/18 | SPSL00000000 | 32 | cc32 | 1 | 1 | 1 | B | 1 | NadA-1 | 5 | 3 | 3-725 | 0 | 0 | 3-3 | 10 | 2 | 2 |
| N.109/18 | SPSN00000000 | 12060 | cc32 | 1 | 1 | 1 | B | 1 | NadA-1 | 5 | 3 | 3-1 | 19 | 15 | 5-1 | 3 | 185 | 2 |
| N.112/18 | SEUM00000000 | 485 | cc41/44 | 4 | 4 | 1 | B | 0 | 0 | 65 | 58 | 3-1 | 7-2 | 4 | 1-5 | 90 | 4 | 18 |
| N.117/18 | SEUN00000000 | 34 | cc32 | 1 | 1 | 1 | B | 1 | NAdA-1 | 334 | 321 | 3-1 | 19 | 15 | 5-1 | 3 | 2 | **257** |
| N.118/18 | SJTS00000000 | 34 | cc32 | 1 | 1 | 1 | B | 1 | NAdA-1 | 334 | 321 | 3-1 | 19 | 15 | 5-1 | 3 | 2 | **257** |
| N.138/18 | SPMT00000000 | 11827 | cc35 | 24 | 24 | 2 | A | 0 | 0 | 19 | 21 | 3-45 | 19 | 15-1 | 5-5 | 3 | 4 | 72 |
| N.145/18 | SPLP00000000 | 461 | cc461 | 47 | 47 | 3 | A | 0 | 0 | **1562** | **1390** | 3-109 | 19-2 | 13-1 | 5-5 | 420 | 2 | 5 |
| N.147/18 | SPLO00000000 | 11827 | cc35 | 24 | 24 | 2 | A | 0 | 0 | 19 | 21 | 3-45 | 19 | 15-1 | 1-7 | 14 | 4 | 72 |
| N.149/18 | SWJK00000000 | 11827 | cc35 | 24 | 24 | 2 | A | 0 | 0 | 19 | 21 | 3-45 | 19 | 15-1 | 1-7 | 14 | 4 | 72 |
| N.154/18 | VHNY00000000 | 461 | cc461 | 47 | 47 | 3 | A | 0 | 0 | **1562** | **1390** | 3-14 | 19-2 | 13-1 | 5-5 | 9 | 2 | 5 |
| N.155/18 | VKJR00000000 | 1768 | na | 218 | 218 | 1 | B | 0 | 0 | 275 | 53 | 3-16 | 5 | 2 | 1-5 | 9 | **314** | 5 |
| N.172/18 | SWJJ00000000 | 11827 | cc35 | 24 | 24 | 2 | A | 0 | 0 | 19 | 21 | 3-45 | 19 | 15-1 | 1-7 | 14 | 4 | **259** |
| N.175/18 | VHNX00000000 | 485 | cc41-44 | 4 | 4 | 1 | B | 0 | 0 | 65 | 58 | 3-1 | 7-2 | 4 | 1-5 | 90 | 4 | 18 |
| N.187/18 | VKJZ00000000 | 34 | cc32 | 1 | 1 | 1 | B | 1 | NadA-1 | 5 | 3 | 3-1 | 19 | 15 | 5-1 | 3 | 2 | 2 |
| N.198/18 | VKJS00000000 | 9184 | cc213 | 544 | 544 | 1 | B | 0 | NadA-4/5 | 33 | 18 | 3-14 | 22 | 14 | 5-5 | 14 | 3 | 34 |
| N.202/18 | VKJT00000000 | 14632 | cc461 | 47 | 47 | 3 | A | 0 | 0 | **1562** | **1390** | 3-14 | 19-2 | 13-1 | 5-5 | 420 | 2 | 5 |
| N.210/18 | SWJL00000000 | 485 | cc41/44 | 4 | 4 | 1 | B | 0 | 0 | 65 | 58 | 3-1 | 7-2 | 4 | 1-5 | 9 | 4 | 18 |
| N.212/18 | VKJU00000000 | 7983 | cc60 | 13 | 13 | 1 | B | 0 | 0 | 15 | 24 | 2-65 | 5 | 2 | 1-7 | 20 | 3 | 4 |
| N.226/18 | VHNU00000000 | 12060 | cc32 | 1 | 1 | 1 | B | 1 | NadA-1 | 5 | 3 | 3-1 | 19 | 15 | 5-1 | 3 | 185 | 2 |
| N.234/18 | SWJM00000000 | 11827 | cc35 | 24 | 24 | 2 | A | 0 | 0 | 19 | 21 | 3-45 | 19 | 15-1 | 1-7 | 14 | 4 | 72 |
| N.242/18 | VKJV00000000 | 12060 | cc32 | 1 | 1 | 1 | B | 1 | NadA-1 | 5 | 3 | 3-1 | 19 | 15 | 5-1 | 3 | 185 | 2 |
| N.246/18 | VKJW00000000 | 34 | cc32 | 1 | 1 | 1 | B | 1 | NadA-1 | 5 | 3 | 3-1 | 19 | 15 | 5-1 | 3 | 2 | 2 |
| N.253/18 | VHNL00000000 | 3327 | cc865 | 119 | 119 | 2 | A | 0 | 0 | 15 | 24 | 3-106 | 21 | 16-36 | 5-8 | 9 | 4 | 40 |
| N.270/18 | VHNM00000000 | 461 | cc461 | 47 | 47 | 3 | A | 0 | 0 | **1604** | **1431** | **3-986** | 19-2 | 13-7 | 5-5 | 420 | 2 | 5 |
| N.273/18 | VHNN00000000 | 11827 | cc35 | 24 | 24 | 2 | A | 0 | 0 | 19 | 21 | 3-45 | 19 | 15-1 | 1-7 | 14 | 4 | 72 |
| N.278/18 | VHNO00000000 | 162 | cc162 | 21 | 21 | 2 | A | 0 | 0 | 11 | 20 | 3-249 | 7-2 | 4 | 5-9 | 22 | 4 | 28 |
| N.281/18 | SWJO00000000 | 11827 | cc35 | 24 | 24 | 2 | A | 0 | 0 | 19 | 21 | 3-45 | 19 | 15-1 | 1-7 | 14 | 4 | 72 |
| N.286/18 | VHNP00000000 | 162 | cc162 | 740 | 740 | 3 | A | 0 | 0 | 11 | 20 | 3-81 | 7-2 | 4 | 5-9 | 22 | 4 | 28 |
| N.289/18 | VHNQ00000000 | 11827 | cc35 | 24 | 24 | 2 | A | 0 | 0 | 19 | 21 | 3-45 | 19 | 15-1 | 1-7 | 14 | 4 | 72 |
| N.300/18 | VHNR00000000 | 461 | cc461 | 47 | 47 | 3 | A | 0 | 0 | **1605** | **1432** | **3-986** | 19-2 | 13 | 1-7 | 420 | 2 | **264** |
| N.302/18 | VHNZ00000000 | 12060 | cc32 | 1 | 1 | 1 | B | 1 | NadA-1 | 5 | 3 | 3-1 | 19 | 15 | 5-1 | 3 | 185 | 2 |
| N.306/18 | VHNS00000000 | **15124** | cc213 | 45 | 45 | 3 | A | 0 | 0 | 33 | 18 | 3-14 | 22 | 14 | 5-9 | **885** | 3 | 34 |

ST = sequence type

na = not assigned

PorA VR1, VR2 = PorA variable region 1 and 2

FetA VR = FetA variable region

0 = isolate lacks a functional allele or absence of gene

bold = newly described sequence;

Vaccine antigen variants reactivity for 4CMenB vaccine according to Muzzi et al [1], “green” highlight, covered by gMATS; “red” highlight, not covered by gMATS; “grey” highlight, unpredictable by gMATS.

Vaccine antigen variants reactivity for MenB-FHbp vaccine according data extracted from MenDeVAR Index (https://pubmlst.org/neisseria/; assessed October 27, 2020) [2]. “green” highlight, exact matches to the sequence variants; “amber” highlight, cross-reactive in experimental studies; “grey” highlight, insufficient data.

1. Muzzi A, Brozzi A, Serino L, Bodini M, Abad R, Caugant D, et al. Genetic Meningococcal Antigen Typing System (gMATS): A genotyping tool that predicts 4CMenB strain coverage worldwide. Vaccine. 2019; 37(7):991–1000. https://doi.org/10.1016/j.vaccine.2018.12.061
2. Rodrigues CMS, Jolley KA, Smith A, Cameron JC, Feavers IM, Maiden MCJ. Meningococcal Deduced Vaccine Antigen Reactivity (MenDeVAR) Index: a Rapid and Accessible Tool that Exploits Genomic Data in Public Health and Clinical Microbiology Applications. bioRxiv 2020.08.18.256834; doi: https://doi.org/10.1101/2020.08.18.256834
